# EVI1 overexpression reprograms hematopoiesis via upregulation of *Spi1* transcription

**DOI:** 10.1038/s41467-018-06208-y

**Published:** 2018-10-12

**Authors:** Edward Ayoub, Michael P. Wilson, Kathleen E. McGrath, Allison J. Li, Benjamin J. Frisch, James Palis, Laura M. Calvi, Yi Zhang, Archibald S. Perkins

**Affiliations:** 1 Department of Pathology and Laboratory Medicine, University of Rochester Medical Center, Rochester, NY 14642 USA; 2 James P. Wilmot Cancer Institute, University of Rochester Medical Center, Rochester, NY 14642 USA; 3 Department of Pharmacology and Physiology, University of Rochester Medical Center, Rochester, NY 14642 USA; 4 Department of Pediatrics and Center for Pediatric Biomedical Research, University of Rochester Medical Center, Rochester, NY 14642 USA; 5 Department of Medicine, University of Rochester Medical Center, Rochester, NY 14642 USA

## Abstract

Inv(3q26) and *t*(3:3)(q21;q26) are specific to poor-prognosis myeloid malignancies, and result in marked overexpression of EVI1, a zinc-finger transcription factor and myeloid-specific oncoprotein. Despite extensive study, the mechanism by which EVI1 contributes to myeloid malignancy remains unclear. Here we describe a new mouse model that mimics the transcriptional effects of 3q26 rearrangement. We show that EVI1 overexpression causes global distortion of hematopoiesis, with suppression of erythropoiesis and lymphopoiesis, and marked premalignant expansion of myelopoiesis that eventually results in leukemic transformation. We show that myeloid skewing is dependent on DNA binding by EVI1, which upregulates *Spi1*, encoding master myeloid regulator PU.1. We show that EVI1 binds to the −14 kb upstream regulatory element (−14kbURE) at *Spi1*; knockdown of *Spi1* dampens the myeloid skewing. Furthermore, deletion of the −14kbURE at *Spi1* abrogates the effects of EVI1 on hematopoietic stem cells. These findings support a novel mechanism of leukemogenesis through EVI1 overexpression.

## Abstract (web summary)

Chr3q26 rearrangements cause overexpression of EVI1 and associate with myeloid neoplasms, but the mechanism behind this association is unclear. Here, using a novel mouse model they show that EVI1 causes premalignant myeloid expansion with suppression of other lineages through upregulation of Spi1/PU.1.

## Introduction

Chromosomal rearrangements at 3q26 are associated with poor-prognosis acute myeloid leukemia (AML)^1^, myelodysplastic syndromes (MDS)^2^, and myeloproliferative neoplasms (MPN)^3^, and cause activation of *MECOM*, a gene that encodes multiple zinc-finger (ZF) transcription factor isoforms, including three isoforms of EVI1. These isoforms, generated via alternative splicing, are nuclear factors harboring two ZF domains. Within the hematopoietic system, *Evi1* expression is restricted to long-term and short-term hematopoietic stem cells (HSCs), and under normal conditions, is thereafter during hematopoiesis transcriptionally silent^4^. Activation of the locus, via either chromosomal rearrangement^5^ or by proviral insertion^6,7^, results in marked overexpression of the protein isoforms^8^.

Within hematopoietic tumors, the association between 3q26 rearrangements and myeloid disease is very high: overexpression of EVI1 is virtually never seen in non-myeloid leukemias or lymphomas. The underlying reason for this specific relationship is unknown, but suggest two possibilities: (i) that myeloid cells are particularly susceptible to the transforming effect of EVI1, or (ii) that the overexpression of EVI1 in hematopoietic stem/progenitor cells (HSPCs) drives cells into the myeloid lineage.

While a number of different mechanisms of action for EVI1 have been suggested (reviewed in ref. ^9^), it is still unclear how it contributes to leukemogenesis. This is in large part due to the lack of model systems that allow studying the mechanism of EVI1-associated leukemogenesis (reviewed in ref. ^9^). Experimental efforts to overexpress *Evi1* in mouse models have yielded mixed results^10–14^. These disparate results suggest that EVI1-induced disease is difficult to model in the mouse, perhaps due to technical issues; they also suggest that EVI1 by itself is not sufficient to induce neoplastic disease. While Yamazaki et al.^14^ previously published an EVI1 transgenic model that genetically mimics the 3q26 human leukemias and underlined the significance of GATA2 enhancer, we report the development of the first *Evi1*-inducible system that allowed us to uncover a mechanism behind EVI1-associated leukemogenesis.

Using this system, *Evi1* induction by doxycycline (DOX) causes a massive perturbation of hematopoietic homeostasis, expanding HSCs, suppressing erythropoiesis and lymphopoiesis, and creating a myeloid-skewed phenotype. Our studies provide insight into the molecular mechanism, as they demonstrate that the myeloid skewing is dependent on DNA binding by EVI1; we further show that EVI1 binds to and upregulates *Spi1*, encoding PU.1, a master regulator of myelopoiesis. Furthermore, the myeloid skewing is dependent on expression of PU.1. These findings support a model wherein EVI1 acts via PU.1 to push hematopoietic HSPCs towards the myeloid lineage, with concomitant suppression of erythropoiesis and lymphopoiesis; this myeloid expansion, eventually progresses to AML.

## Results

### Establishment of a mouse model for 3q26 translocations

**Fig. 1:**
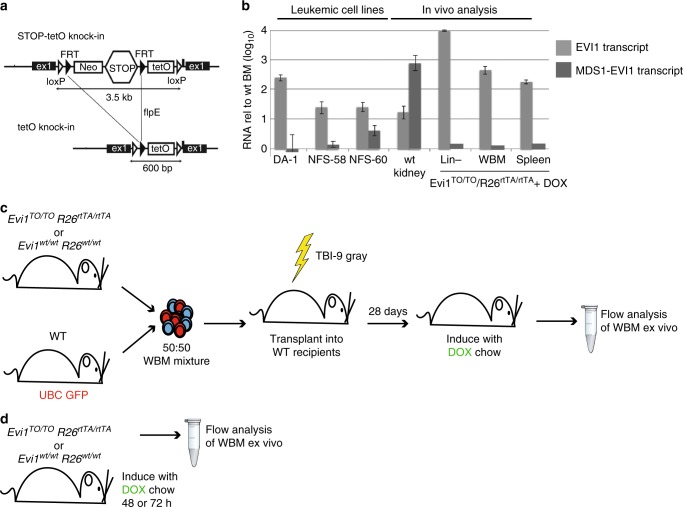
*Evi1*^*TO*^ mouse model for 3q26-rearranged myeloid malignancies. **a** Schematic diagram of the Neo-Stop-Tet Operon (NSTO) construct from Tanaka et al.^63^ that was inserted into the endogenous *Evi1* locus by homologous recombination. The construct consists of a neomycin resistance gene and transcriptional Stop cassette flanked by FRT sites, followed by seven Tet operons in succession and a minimal CMV promoter. Following homologous recombination in embryonic stem cells, the Neo-STOP cassette was removed by induction of flpE recombinase. Black triangles represent FRT sites, and white triangles represent loxP sites. **b** Reverse transcription-quantitative polymerase chain reaction (RT-qPCR) analysis of RNA from cultured leukemic cell lines bearing a provirally-activated EVI1 allele (left-hand columns), or RNA from mouse tissues from wild-type (kidney, an organ with relatively high levels of expression of both *Evi1* and *Mds1-Evi1*^68^), and from DOX-induced *Evi1*^*TO*^, *Rosa26*^*rtTA*^ mice. Shown is quantitation of *Evi1* and *Mds1-Evi1* transcripts. Data is normalized to EVI1 RNA levels in WT bone marrow; log_10_ scale. **c** Schematic of competitive transplant long-term induction model. CD45.2 whole bone marrow from either *Evi1*^*TO*^ or a WT control with the indicated genotypes was mixed at a 1:1 ratio with WT UBC-GFP-positive bone marrow and transplanted into lethally irradiated recipient mice. Mice were placed on DOX chow at 4 weeks post transplant and analyzed 2, 6, or 10 weeks post induction. Long-term induction experiment was done twice (set A, *n* = 24, set B, *n* = 24). Each time point of each set had 8 mice (WT, *n* = 4, EVI1, *n* = 4). (Total *n* = 48). **d** Schematic of endogenous short-term induction model. Mice were induced with DOX chow for 48 or 72 h and whole bone marrow was harvested for ex vivo analysis

To mimic the effects of rearrangements at 3q26 in AML, we created a tetracycline (Tet)-inducible allele of *Evi1* (termed *Evi1*^*TO*^) in the mouse by inserting seven Tet operons within the first exon (Fig. 1a^15^), allowing for the induction of all three isoforms of *Evi1*^8,9^. *Evi1*^*TO/TO*^ mice are viable and fertile without phenotype indicating that the allele functions normally in the uninduced state. Through genetic crossings, we then introduced the ubiquitously-expressed^16–18^ reverse-Tet transactivator (rtTA) allele (*Rosa26*^*rtTA/rtTA*^; ref. ^19^).

To assess whether *Evi1* can be induced in vivo, we tested induction in mice harboring *Evi1*^*TO/TO*^/*Rosa26*^*rtTA/rtTA*^ alleles: treatment of these mice with DOX results in dramatic upregulation of the major *Evi1* transcript in hematopoietic cells to levels seen in leukemic cells (Fig. 1b); two other mRNA transcripts, encoding shorter isoforms, were also upregulated by DOX (Supplementary Figure 1); however, the longer, MDS1-EVI1 mRNA transcript, (encoding PRDM3, the PR-domain-containing isoform), was not upregulated (Fig. 1b). This accurately models the pattern of *MECOM* expression commonly seen in human AML with 3q26 rearrangements, in which the EVI1 isoforms are upregulated, but typically not the longer, MDS1-EVI1/PRDM3 isoforms; this particular expression pattern is associated with the poorest prognosis^20^.

To assess the effect of EVI1 overexpression on hematopoiesis in a context that mimics human myeloid malignancy, in which the oncogene-expressing cells are mixed in with normal hematopoietic cells, we performed competitive transplantations in which the oncogene-expressing bone marrow was mixed 1:1 with WT bone marrow (Fig. 1c); for examination of the short-term effects of EVI1 overexpression, we also fed DOX chow directly to the *Evi1*^*TO/TO*^/*Rosa26*^*rtTA/rtTA*^ mice themselves (Fig. 1d).

In the competitive transplantation model, to allow each donor origin to be traced by flow cytometry, the WT competitor was marked with UBC-GFP, while *Evi1*^*TO/TO*^/*Rosa26*^*rtTA/rtTA*^ cells were of CD45.2/GFP-negative origin; transplants were performed into lethally irradiated CD45.1/GFP-negative recipients. For a control cohort, we transplanted *Evi1*^*WT/WT*^/*Rosa26*^*WT/WT*^ CD45.2/GFP-negative bone marrow cells instead of the *Evi1*^*TO/TO*^/*Rosa26*^*rtTA/rtTA*^ cells together with GFP-positive WT cells (Fig. 1c). We documented that the stem cell and progenitor populations from the bone marrow of *Evi1*^*TO/TO*^/*Rosa26*^*rtTA/rtTA*^ donors did not differ from that of the control just prior to transplantation (Supplementary Figure 2A). Recipients were analyzed for the engraftment of donors’ BM 4 weeks post transplantation and prior to EVI1 induction, which showed that the two donors (GFP-positive and GFP-negative) were no different, both in control recipients and those receiving *Evi1*^*TO/TO*^/*Rosa26*^*rtTA/rtTA*^ cells (Supplementary Figure 2B, left). After confirming successful engraftments, recipients were placed on DOX chow to induce *Evi1*. After 10 weeks, we analyzed cells in peripheral blood and bone marrow. The mice appeared healthy, and CBCs revealed no cytopenias (not shown). We performed flow analysis of marrow and blood, identifying *Evi1*^*TO/TO*^/*Rosa26*^*rtTA/rtTA*^ cells by lack of GFP, and engraftment ratio was determined, which revealed a significant (more than twofold) expansion of the EVI1-overexpressing compartment (Supplementary Figure 2B, right).

### EVI1 overexpression suppresses erythropoiesis

**Fig. 2:**
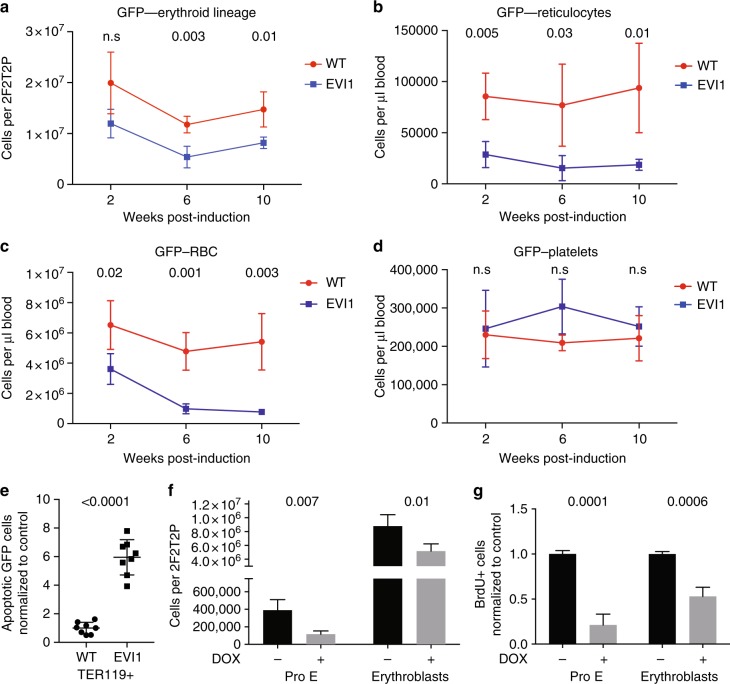
EVI1 overexpression suppresses erythropoiesis. **a** Quantification of erythroid (TER119+) cells overexpressing EVI1 (GFP−) in bone marrow of competitively transplanted recipients harvested 2, 6, or 10 after DOX induction. (WT *n* = 4 mice, EVI1 *n* = 4 mice at each time point, total *n* = 24). **b**–**d** Numbers of donor-derived reticulocytes (GFP−, Ter119+, CD71+), erythrocytes (GFP−,Ter119+, CD71−), and platelets (GFP−, low SSC/FSC, CD41+) as indicated at 2, 6, and 10 weeks post induction per microliter of peripheral blood of mice competitively transplanted with DOX-induced *Evi1*^*TO*^, *Rosa26*^*rtTA*^ mice or WT cells. (WT *n* = 4 mice, EVI1 *n* = 4 mice at each time point, total *n* = 24). **e** Quantification of apoptotic (annexin-v+ 7AAD−) erythroid (TER119+) cells overexpressing EVI1 (GFP−) or WT WBM of competitively transplanted recipients, expressed relative to WT GFP− cells from the control transplant, harvested 10 weeks after DOX induction. Each dot represents an individual recipient mouse. (WT = 8 mice, EVI1 = 8 mice). **f**, **g** Quantification of total (**f**) and BrdU-positive (**g**) proerythroblasts (TER119+^low^ cKit+), and erythroblasts (TER119+ CD71+) in WBM of *Evi1*^*TO/TO*^ *Rosa26*^*rtTA/rtTA*^ mice harvested 48 h with or without DOX induction. (WT *n* = 4, EVI1 *n* = 4). For all panels, error bars represent standard deviation; *p* values calculated with Student’s *t*-test

Analysis of the erythroid lineage in competitively transplanted mice at 2, 6, and 10 weeks post induction showed a deficiency in the ability of the EVI1-overexpressing cells to effectively contribute to erythropoiesis (Fig. 2a–c). At the 6 and 10 weeks post-induction time points, the number of EVI1-overexpressing Ter119-positive nucleated erythroid cells within the bone marrow was significantly lower than the number of GFP-negative WT cells in control transplant recipients (Fig. 2a). In the peripheral blood of chimeric mice harboring *Evi1*^*TO/TO*^, *Rosa26*^*rtTA/rtTA*^ cells, EVI1-overexpressing reticulocyte, and erythrocyte numbers were significantly lower than control at every time point (Fig. 2b, c). During the same time period, there was no difference in platelets (Fig. 2d), or megakaryocytes between EVI1-overexpressing and wild-type GFP cells.

To understand the etiology of the lower numbers of erythroid cells in the EVI1-overexpressing compartment, we quantitated apoptosis and proliferation in erythroid progenitors using flow cytometry; these studies revealed a sixfold increase in apoptosis within the erythroblasts relative to WT cells (Fig. 2e), and a marked drop in the number of, and proliferation of, both proerythroblasts and erythroblasts relative to WT (Fig. 2f, g).

In summary, EVI1 overexpression has a dramatic effect on erythropoiesis and this is mediated by both an increase in apoptosis and a decrease in proliferation.

### EVI1 overexpression suppresses lymphopoiesis

**Fig. 3:**
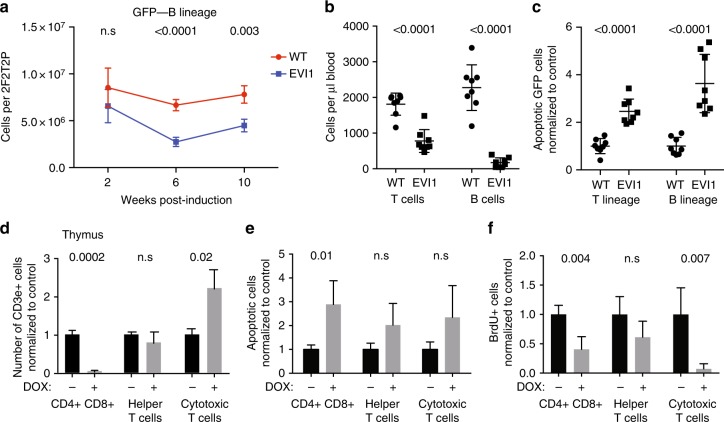
EVI1 overexpression suppresses lymphopoiesis. **a** Quantification of B lymphocytes (B220+) cells overexpressing EVI1 (GFP−) and WT control (GFP−) in WBM of competitively transplanted recipients harvested 2, 6, or 10 after DOX induction. (WT *n* = 4 mice, EVI1 *n* = 4 mice at each time point, total *n* = 24). **b** Circulating donor-derived T cells (GFP−, CD3e+, CD11b−) and B cells (GFP−, CD19+, CD11b−) in competitively transplanted mice at 10 weeks post induction. Each time point (WT *n* = 8 mice, EVI1 *n* = 8 mice). **c** Quantification of apoptotic (annexin-V+, 7AAD−) T lymphocytes (CD3e+) and B lymphocytes (B220+) overexpressing EVI1 (GFP−) compared to WT (GFP−) in WBM of competitively transplanted recipients harvested 10 weeks after DOX induction. Each dot represents an individual recipient mouse. (WT *n* = 8 mice, EVI1 *n* = 8 mice). **d** Quantification of CD4/CD8 double-positive cells (CD3e+, CD4+, CD8a+), helper T cells (CD3e+, CD4+, CD8a−), and cytotoxic T cells (CD3e+, CD4-, CD8a+) in thymus of *Evi1*^*TO/TO*^ *Rosa26*^*rtTA/rtTA*^ mice harvested 48 h with or without DOX induction. (WT *n* = 4, EVI1 *n* = 4). **e** Quantification of apoptotic (annexin-v+ 7AAD−) CD4/CD8 double-positive cells (CD3e+, CD4+, CD8a+), helper T cells (CD3e+, CD4+, CD8a−), and cytotoxic T cells (CD3e+, CD4−, CD8a+) in thymi of *Evi1*^*TO/TO*^ *Rosa26*^*rtTA/rtTA*^ mice harvested 48 h with or without DOX induction. (WT *n* = 4, EVI1 *n* = 4). **f** Quantification of BrdU+ CD4/CD8 double-positive cells (CD3e+, CD4+, CD8a+), helper T cells (CD3e+, CD4+, CD8a−), and cytotoxic T cells (CD3e+, CD4−, CD8a+) in thymi of *Evi1*^*TO/TO*^ *Rosa26*^*rtTA/rtTA*^ mice harvested 48 h with or without DOX induction. (WT *n* = 4, EVI1 *n* = 4). For all panels, error bars represent standard deviation; *p* values calculated with Student’s *t*-test

We examined the bone marrow at 2–10 weeks post induction for changes in T and B cells; we noted significantly lower numbers of EVI1-overexpressing B-cell lineage cells within the bone marrow at 6 and 10 weeks time points (Fig. 3a). While the number of *Evi1*^*TO/TO*^/*Rosa26*^*rtTA/rtTA*^ T cells in the bone marrow was not significantly different from the WT control, examination of blood for lymphocytes showed a marked diminution in both T and B cells from the EVI1-overexpressing compartment: at 10 weeks post induction, EVI1 overexpression caused a decrease in peripheral T cells from ≈1800 cells per μl to ≈750 cells per μl, and nearly eliminated the peripheral B cells completely (Fig. 3b). This was associated with a two to threefold increase in the number of apoptotic bone marrow T and B cells (Fig. 3c).

We found that the number of T cells in the marrow was low, which is to be expected: T cells are derived from HSCs in the bone marrow, but at an early stage, migrate from the bone marrow to the thymus, where they complete their development. To better assess the effect of EVI1 overexpression on T cells, we enumerated T cells in the thymus at the double (CD4/CD8)-positive stage, and at the CD4 or CD8 single-positive stage. This revealed a marked diminution of double-positive cells relative to control but little change in CD4-positive T cells (Fig. 3d). Interestingly, there was an increase in CD8-positive T cells (Fig. 3d). To further assess these changes, we assessed apoptosis and proliferation (Fig. 3e, f) and found increased apoptosis only in the double-positive compartment, but decreased proliferation in both the double-positive and CD8 single-positive population.

### EVI1 overexpression expands myelopoiesis

**Fig. 4:**
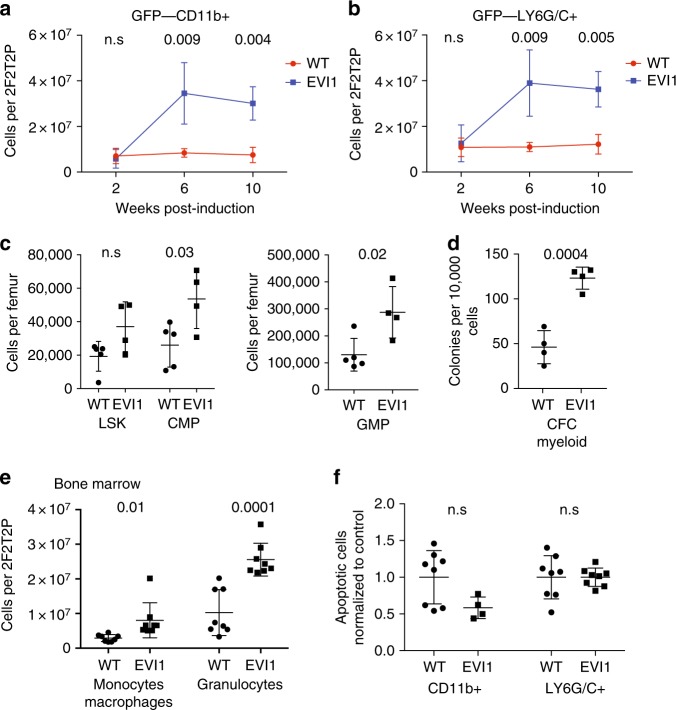
EVI1 overexpression expands myelopoiesis. **a** Quantification of GFP−, CD11b+ myelocytes, either overexpressing EVI1 or WT as indicated in WBM of competitively transplanted recipients, harvested 2, 6, or 10 weeks after DOX induction. (WT *n* = 4 mice, EVI1 *n* = 4 mice at each time point, total *n* = 24). **b** Quantification of GFP− LY6G/C+ myelocytes, either overexpressing EVI1 or WT in WBM of competitively transplanted recipients harvested 2, 6, or 10 weeks after DOX induction. (WT *n* = 4 mice, EVI1 *n* = 4 mice at each time point, total *n* = 24). **c** Flow cytometry analysis of progenitor populations from recipient mice after 10 weeks of DOX treatment. Data represents mean number of cells per femur for individual mice. (WT *n* = 5, Evi1+ *n* = 4). **d** Relative numbers of GFP− CFC-myeloid (G−, M and GM-CFC) in bone marrow of competitively transplanted mice 10 weeks post induction, comparing the *Evi1*^*TO/TO*^ *Rosa26*^*rtTA/rtTA*^:WT to WT:WT transplants as indicated. (WT *n* = 8, EVI1 *n* = 8). **e** Quantification of GFP− monocyte/macrophage (nucleated, CD11b+, Gr1−) or GFP− granulocytes (nucleated, CD11b+, Gr1+) as determined by imaging flow cytometry in bone marrow of competitively transplanted mice, comparing *Evi1*^*TO/TO*^ *Rosa26*^*rtTA/rtTA*^ donor cells to WT donor cells, 10 weeks post induction. (WT *n* = 8, EVI1 *n* = 8). **f** Quantification of GFP− apoptotic (annexin-V+ 7AAD−) CD11b+ myelocytes (left) and LY6G/C+ myelocytes (right), comparing WT to EVI1-overexpressing cells in WBM of competitively transplanted recipients harvested 10 weeks after DOX induction. Each dot represents an individual recipient mouse. (WT *n* = 8, EVI1 *n* = 8). For all panels, error bars represent standard deviation; *p* values calculated with Student’s *t*-test

At 2 weeks post induction, EVI1-overexpressing and control bone marrows showed the same number of myeloid cells (GFP-negative, CD11b-positive (Fig. 4a) or Ly6G/C-positive (Fig. 4b)). However, at 6 and 10 weeks post induction, there was marked expansion of the EVI1-overexpressing myeloid compartment, from ≈7.5e6 cells/2F2T2P to ≈30e6 cells/2F2T2P for CD11b+ cells, and from ≈12e6 cells/2F2T2P to ≈36e6 cells/2F2T2P for LY6G/C+ cells (Fig. 4a). We also quantified HSCs and myeloid progenitors using flow cytometry at 10 weeks post induction; The expansion of lineage-negative, c-Kit+, Sca-1+ (LSK cells; approximately twofold) was not significant (*p* > 0.05), but both CMPs and granulocyte-monocyte progenitors (GMPs) were significantly increased (Fig. 4c). By performing colony-forming assay, we found that myeloid colony-forming cells were over 2.5-fold increased relative to controls (Fig. 4d). By flow-based analysis, monocytes/macrophages and granulocytes within the marrow were increased (Fig. 4e). However, blood monocytes were unchanged, and granulocytes were increased but not as significantly as in the marrow. Analysis of annexin-V revealed no increase in apoptosis of the marrow myeloid cells (Fig. 4f). We also assessed the composition of the LSK compartment in the marrows, which contain HSCs and multipotent progenitors (MPPs) using the surface marker phenotype described and detailed by Cabezas-Wallscheid et al.^21^ (Supplementary Figure 2C, Supplementary Table 1). We found that Evi1 expression expands HSCs, multipotent MPP2s, and the myeloid-biased MPP3 populations, while reducing the erythroid-biased MPP4 populations; no change in MPP1 was seen (Supplementary Figure 2D).

In summary, these data indicate that *Evi1* overexpression induces myeloid expansion via a proliferative and a survival advantage; increased maturation/differentiation from uncommitted progenitors at a higher rate may also play a role.

### EVI1 overexpression results in AML

**Fig. 5:**
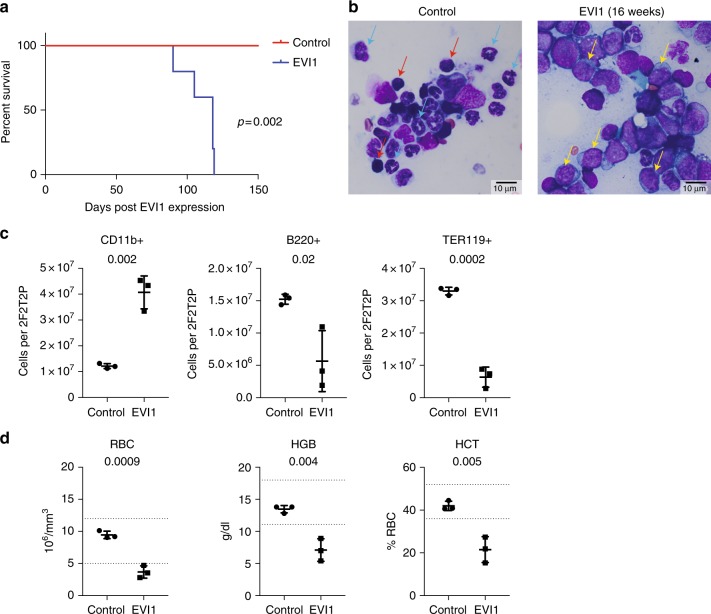
EVI1 overexpression results in AML with intermediate latency. **a** Kaplan–Meier curve showing survival of CD45.1 mice that were transplanted with *WT*:*Evi1*^*TO/TO*^, *R26R*^*rtTA/rtTA*^ at a 1:1 ratio with and without DOX induction. Mice were harvested when morbidity presented (survival range = 90–119 days, median = 118 days, *n* = 5) **b** Representative Wright-Giemsa-stained bone marrow brush smears from CD45.1 mice that were transplanted with *WT*:*Evi1*^*TO/TO*^, *R26R*^*rtTA/rtTA*^ at a 1:1 ratio without (left) or with (right) DOX induction at the day of harvest. Mice were harvested when morbidity presented. Blue arrows, maturing myeloid forms (metamyelocytes and bands); red arrows, erythroblasts; yellow arrows, leukemic blasts (scale bars: 10 μm). **c** Quantification of CD11b+ cells (left), B220+ cells (middle), or TER119+ cells (right) using flow cytometry analysis on WBM of CD45.1 mice that were transplanted with *WT*:*Evi1*^*TO/TO*^, *R26R*^*rtTA/rtTA*^ at a 1:1 ratio with and without DOX induction at the day of harvest. Mice were harvested when morbidity presented (*n* = 3). **d** Quantification of red blood cells (RBC), hemoglobin (HGB), and hematocrit (HCT) using complete blood count assay performed on PB from CD45.1 mice that were transplanted with *WT*:*Evi1*^*TO/TO*^, *R26R*^*rtTA/rtTA*^ at a 1:1 ratio with and without DOX induction at the day of harvest. Mice were harvested when morbidity presented (*n* = 3). For all panels, error bars represent standard deviation; *p* values calculated with Student’s *t*-test

To determine if chronic overexpression of EVI1 might result in leukemia, we aged a cohort of five mice, competitively transplanted with a 1:1 mix of WT bone marrow cells:*Evi1*^*TO/TO*^/*Rosa26*^*rtTA/rtTA*^ cells, chronically on DOX chow to maintain high-level expression of EVI1. All five mice succumbed, becoming moribund at 90–119 days of DOX treatment (median = 118 days; Fig. 5a; *p* = 0.002; Fig. 5a); flow and morphological analysis revealed AML in all mice analyzed, with the bone marrow replete with blast forms (Fig. 5b), which, by immunophenotyping by flow cytometry, were positive for Mac1 (CD11b) (Fig. 5c). The peripheral blood revealed severe anemia (Fig. 5d).

### EVI1 myeloid-biased phenotype depends on DNA binding by EVI1

**Fig. 6:**
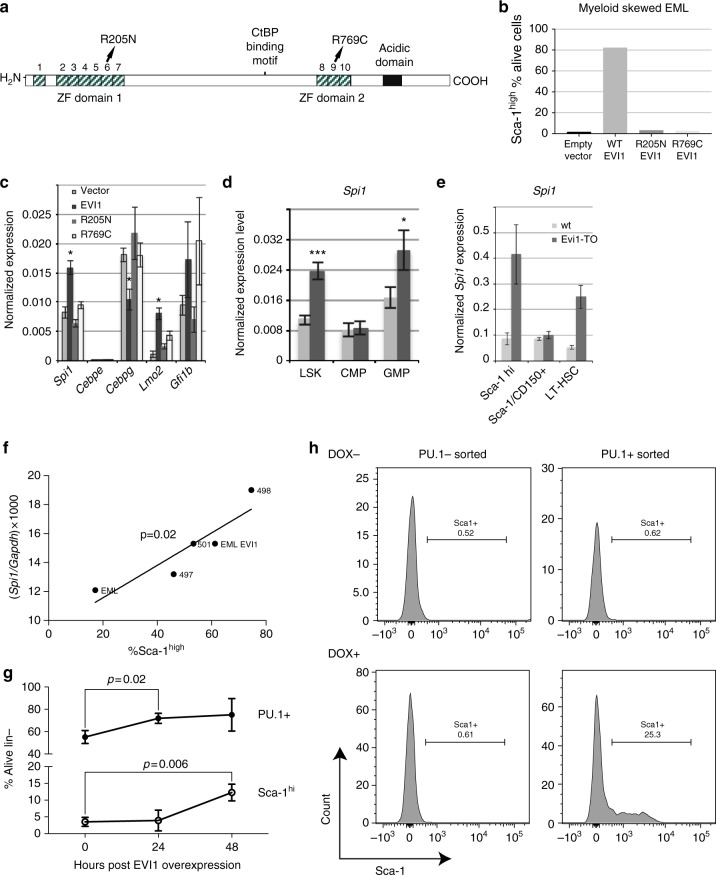
EVI1-induced myeloid skewing: downstream effectors include PU.1. **a** Schematic of EVI1 protein domains, documenting the two zinc-finger domains and the location of the two arginine mutations that disrupt ZF1 and ZF2. **b** Flow cytometric quantitation of Sca-1^hi^ myeloid-skewed population in EML cells transduced with the indicated retroviral construct, designated on *x* axis. **c** RT-qPCR analysis of RNA from sorted Sca-1^hi^ EML cells transduced with the indicated constructs. Gene names are indicated on the *x* axis. All qPCR reactions performed in triplicate; displayed values represent the average. Errors bars represent standard deviation between qPCR reactions. *p < 0.005. **d** RT-qPCR data (normalized to *Gapdh*) showing expression of *Spi1* in progenitor populations from *Evi1*^*TO/TO*^*R26r*^*tTA/rtTA*^ CD45.2 donor compartments at 10 weeks of induction. **e** RT-qPCR data (normalized to *Gapdh*) for *Spi1* expression in sorted bone marrow progenitor populations from either WT mice or *Evi1*^*TO*^, *R26*^*rtTA*^ mice (as indicated) treated with DOX for 72 hrs. **f** Scatterplot comparing *Spi1* mRNA expression (*y* axis) to percent Sca-1^hi^ cells (*x* axis) for five EML cell populations: unmanipulated EMLs (“EML”); EVI1-overexpressing EMLs (“EML EVI1”), and three derivatives of the latter, transduced with either a *Spi1*-specific shRNA (497, 501) or a control shRNA (498). The *p* value is calculated using linear regression analysis. **g** Flow cytometric analysis for Sca-1 and PU.1 (GFP+; top, percent positive) and Sca-1 (bottom; percent positive) on lineage-subpopulation within WBM harvested from *Evi1*^*TO/TO*^, *Rosa26*^*rtTA/rtTA*^, *Spi1*^*GFP/GFP*^ mice at 0, 24, or 48 h after DOX induction (0 h *n* = 3, 24 h *n* = 3, 48 h *n* = 3). **h** Flow cytometric analysis for Sca-1 in Lin-Sca-1-cKit-PU.1GFP-sorted (left two histograms), or Lin-Sca-1-cKit-PU.1GFP+sorted (right two histograms), uninduced (top) or induced with 1 μg/ml DOX (bottom) for 72 h. Error bars represent standard deviation; *p* values for all except *F* were calculated using Student’s *t*-test

The data shown above indicate that *Evi1* overexpression results in suppression of erythroid and lymphoid cells, and expansion of myeloid cells. Since EVI1 harbors two DNA-binding zinc-finger domains (Supplementary Figure 1G; refs. ^22,23^) and can modulate the transcription of target genes via binding in cis (e.g., refs. ^15,24^), we wished to determine if the myeloid skewing phenotype induced by EVI1 was dependent on its binding to DNA using two EVI1 mutants (Fig. 6a): EVI1-R205N, which lacks DNA binding via ZF domain1^8^ and EVI1-R769C, which lacks DNA binding via ZF2^25^. However, rather than testing these in our mouse model that would have entailed the development of two new mouse strains, instead we utilized EML cells^26^, a hematopoietic progenitor cell line that does not express *Evi1*. Previous analysis of EML cells^27,28^ indicated that Sca-1 provides an excellent marker for the lineage proclivity of these progenitor cells, with Sca-1^lo^ cells showing high GATA-1 expression and being poised for erythroid differentiation, and Sca-1^hi^ cells expressing high PU.1 expression and being poised for myeloid differentiation^27,28^. To determine if Sca-1 upregulation depended on the action of EVI1 on downstream targets, we tested the two EVI1 DNA-binding mutants (Fig. 6a, EVI1-R205N and EVI1-R769C) in EML cells for their ability to upregulate Sca-1. Both of these mutations abrogated the upregulation of Sca-1 (Fig. 6b), indicating that the myeloid skewing phenotype requires both ZF1 and ZF2 domains and that the action of EVI1 on downstream target genes is an essential mechanistic link. To investigate this further, we assessed the expression of five key hematopoietic regulators in vector-transduced EMLs, compared to EVI1-expressing EMLs, as well as those expressing ZF mutants, specifically focusing on the Sca-1^hi^ population (Fig. 6c). These regulators were *Spi1*, encoding PU.1, a major regulator of the lineage choice between erythroid and myeloid cells^29,30^; *Cebpe*, encoding C/EBP-ε, essential for terminal myeloid maturation^31^; *Cebpg*, encoding CEBP-γ, essential for NK cell development^32^ and which interacts with CEBP-α, a major regulator of myelopoiesis^33^; *Lmo2*, encoding a transcriptional regulator and facilitator of DNA replication^34^ that is essential for adult hematopoiesis^35^; and *Gfi1b*, which is required for erythropoiesis and thrombopoiesis^36^. We found significant changes in three of these: *Spi1* (up twofold), *Cebpg* (down 50%), and *Lmo2* (up 10-fold). For *Spi1*, *Cebpg*, and *Lmo2*, EVI1-induced changes in expression were dependent on DNA binding, since point mutations in ZF1 or ZF2 abrogated the effect (Fig. 6c).

### EVI1 myeloid-biased phenotype is dependent on *Spi1*

The transcription factor PU.1 is a master regulator of early myelopoiesis, with highest levels being expressed in CD11b-positive myeloid cells, including macrophages, monocytes, and immature granulocytes, and lower levels in B lymphocytes, while erythroid cells lack expression^37^. We posited that activation of *Spi1* by EVI1 could help explain the expansion of the myeloid compartment in EVI1-overexpressing bone marrow. Thus, we decided to study the link between EVI1, *Spi1*/PU.1, and myeloid skewing.

To confirm the upregulation of *Spi1* by EVI1 overexpression in HSPCs in vivo, we assessed changes in *Spi1* gene expression following EVI1 induction with DOX in our *Evi1*^*TO*^*/Rosa26*^*rtTA*^ mice. We found that EVI1 upregulates *Spi1* expression in HSCs and progenitors (LSK) and in GMPs (Fig. 6d) suggesting that EVI1 increases the expression of the key myeloid gene *Spi1* in early hematopoietic and myeloid progenitor cells. We then analyzed *Spi1* expression in sub-compartments within the LSKs: we observed strong upregulation of *Spi1* within the lineage-negative/Sca-1^hi^ compartment, as well as in long-term HSCs (LT-HSCs; Fig. 6e).

Our data in the EML cell system (Fig. 6b) implies that EVI1 upregulates PU.1, leading to increase in myeloid poised Sca-1^hi^ cells. This predicts that knockdown of PU.1 in EVI1-transduced cells would lead to decreased percentage of Sca-1 high cells. We introduced *Spi1*-specific shRNA constructs specific into EML-EVI1 cells, and quantitated both Sca-1 and *Spi1*; the results show a strong positive correlation between Sca-1 expression and *Spi1* expression: the higher the level of PU.1, the greater the percentage of Sca-1^hi^ cells (Fig. 6f). These data indicate that EVI1-mediated upregulation of Sca-1, and hence myeloid skewing, depends on *Spi1*/PU.1.

### PU.1 is necessary for EVI1-mediated upregulation of Sca-1

To better understand the relationship between EVI1, PU.1, and Sca-1, we turned to an in vivo system in which PU.1 expression can be followed at the single cell level: we utilized a *Spi1*^*gfp*^ allele, in which GFP is driven by the endogenous *Spi1* promoter^37^. We crossed our *Evi1*^*TO*^ and Rosa26^*rtTA*^ alleles with the *Spi1*^*gfp*^ allele and assessed whether DOX-induced upregulation of *Evi1* expression would lead to increased *Spi1*/PU.1 and Sca-1 expression within the bone marrow.

Bone marrow from DOX-induced *Evi1*^*TO*^*/Rosa26*^*rtTA*^*/Spi1*^*gfp*^ mice was isolated at 24 h, and 48 h post induction and analyzed for GFP and Sca-1 expression. We found a significant upregulation of GFP, from 55 to 70% of cells by 24 h (*p* = 0.02) (Fig. 6g). This was followed by a marked increase in Sca-1 expression at 48 h (Fig. 6g, bottom panel).

We wondered whether EVI1 overexpression within early erythroid cells could induce Sca-1 expression. From previous studies^37^, maturing erythroid cells are negative for PU.1. To test this, we isolated lin-negative, c-Kit-negative, Sca-1-negative bone marrow cells from *Evi1*^*TO*^*/Rosa26*^*rtTA*^*/Spi1*^*gfp*^ mice, and separated GFP-negative erythroid progenitors from GFP-positive progenitors. Each population was cultured in vitro and induced with DOX to determine the effects of EVI1 on both PU.1 and Sca-1 expression. Remarkably, Sca-1 induction was confined specifically to the GFP-positive population, indicating that PU.1 is required together with EVI1 to induce Sca-1 expression (Fig. 6h). Interestingly, we did not observe induction of GFP expression in either compartment with DOX treatment.

### EVI1 binds to −14 kb *Spi1* upstream regulatory element

**Fig. 7:**
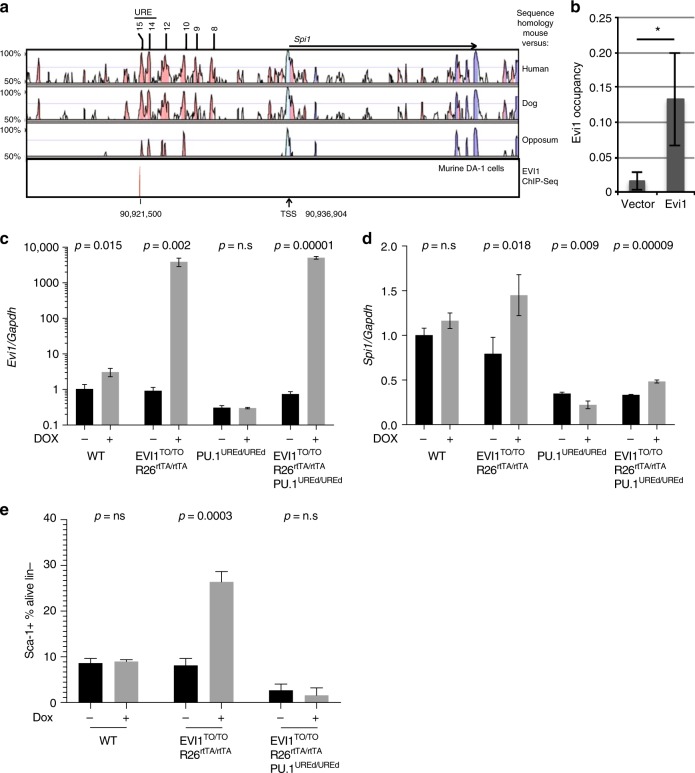
EVI1 binds to the −14kbURE of Spi1 and requires this element for Sca-1 upregulation. **a** Region of mouse genome encompassing the transcription start site (TSS) of *Spi1* together with 25 kb of upstream DNA. Top three bands show peaks of sequence homology between mouse and human, dog, and opossum, as indicated, while the bottom band displays our ChIP-Seq for EVI1 occupancy in the murine myeloid leukemia cell line, DA-1, at −15.45 kb from the *Spi1* transcription start site. Also indicated is the location of the −15 to −14 kb upstream regulatory element, referred to in the text as −14kbURE. **b** ChIP-qPCR data showing EVI1 occupancy at the *Spi1*–15.45 binding site in EML cells transduced with WT EVI1 or vector control. **c**, **d** RT-qPCR for *Evi1* (**c**) or *Spi1* (**d**) on Lin− bone marrow cells, with or without in vivo DOX treatment for 72 h from four genotypes of mice, harboring either *Evi1*^*TO/TO*^ *Rosa26*^*rtTA/rtTA*^ or *Spi-1*^*UREd/UREd*^ genotypes, as indicated. **e** Quantification of Sca-1+cells of WBM from mice with genotypes as indicated either with or without DOX treatment for 72 h (*n* = 3). For all panels, error bars represent standard deviation; *p* values calculated with Student’s *t*-test

Our data indicate a role for both ZF1 and ZF2 of EVI1 in myeloid skewing and in the upregulation of key myeloid regulator, *Spi1*. To further examine the role of EVI1 in transcriptional regulation of *Spi1*, we performed Chromatin Immunoprecipitation Sequencing (ChIP-Seq) on DA.1 cells, an EVI1-overexpressing AML. These data (reported in toto previously^38^), identified a binding site at −15.45 kb from the *Spi1* transcription start site (Fig. 7a), within a previously described regulatory element termed the −14 kb upstream regulatory element (−14kbURE)^39^. We confirmed this binding using ChIP-PCR (Fig. 7b).

### Sca-1 upregulation depends on EVI1 binding to −14 kb *Spi1* URE

To test the role of the −14kbURE in EVI1-mediated upregulation of *Spi1*/PU.1 and Sca-1, we combined the *Evi1*^*TO*^/Rosa26^*rtTA*^ alleles with the *Spi1*^*–14UREdel*^ (ref. ^40^; a deletion that encompasses the −15.45 kb EVI1-binding element), with the expectation that in mice lacking the −14kbURE, EVI1 would not be able to bind to the *Spi1* promoter, which would abrogate EVI1-mediated upregulation of PU.1; this would allow us to assess the requirement for PU.1 in EVI1-mediated upregulation of Sca-1. We generated *Evi1*^*TO*^/Rosa26^*rtTA*^/*Spi1*^*–14UREdel*^ and *Evi1*^*TO*^/Rosa26^*rtTA*^ mice, and then treated these (as well as WT and *Spi1*^*–14UREdel*^ control mice) with DOX (or not) for 72 h; bone marrow was analyzed for upregulation of EVI1, *Spi1*/PU.1, and Sca-1. As expected, administration of DOX resulted in upregulation of EVI1 in both groups harboring the *Evi1*^*TO*^ and *Rosa26*^*rtTA*^ alleles; there was no induction in WT or *Spi1*^*–14UREdel*^ mice (Fig. 7c). DOX treatment resulted in an 83% increase in *Spi1*/PU.1 expression in *Evi1*^*TO*^/Rosa26^*rtTA*^ mice, but no change in WT or *Spi1*^*–14UREdel*^ mice; however, baseline levels of *Spi1*/PU.1 transcripts were lower in the latter (Fig. 7d; ref. ^40^). In *Evi1*^*TO*^/Rosa26^*rtTA*^/*Spi1*^*–14UREdel*^ mice, we did observe a DOX-induced increase in *Spi1*/PU.1 transcripts of 45% despite the deletion of the −14kbURE; while this is less than the increase seen in *Evi1*^*TO*^/Rosa26^*rtTA*^ mice, the increase of 45% was still significant (*p* < 0.001), indicating that EVI1-responsive elements exist outside of the −14kbURE.

Regarding Sca-1 expression: as expected, DOX treatment of *Evi1*^*TO*^/Rosa26^*rtTA*^ mice resulted in a more than threefold increase in Sca-1-expressing lineage-negative bone marrow cells (from 8 to 29%), while in *Evi1*^*TO*^/Rosa26^*rtTA*^/*Spi1*^*–14UREdel*^ mice, induction of EVI1 resulted in no change in Sca-1 expression (both <5% Sca-1+; Fig. 7e, Supplementary Figure 3). These data indicate that EVI1-induced upregulation of Sca-1 is dependent on upregulation of *Spi1*/PU.1 by EVI1 via the −14kbURE.

## Discussion

Overexpression of EVI1 is associated with poor-prognosis AML with a median overall survival of 12 months^41^. Despite much investigation, the mechanisms by which EVI1 contributes to leukemia have remained obscure. One key observation is that EVI1 is associated specifically with myeloid malignancies^42–44^. Our findings here offer an insight into this curious association: EVI1 acts through the activation of PU.1 to promote myeloid expansion and interfere with erythropoiesis and lymphopoiesis. Our data indicate that EVI1 overexpression does not by itself cause immediate malignant transformation of hematopoietic cells, but regulates the functional output of the hematopoietic system. Importantly, our analysis also indicates that EVI1 overexpression does not preclude terminal differentiation of the myeloid cells: the EVI1-overexpressing myeloid cells are well-represented in the peripheral blood, and by morphology, these appear normal, consistent with previous findings^45^.

This model mimics human myeloid malignancies bearing rearrangements at 3q26 for the following reasons: (1) the native, unmutated gene, with all of the alternatively spliced isoforms, is upregulated (Supplementary Figure 1H); (2) the levels of EVI1 expression are similar to activated *MECOM* alleles (Fig. 1b); (3) because we induce EVI1 in the transplanted setting, only hematopoietic cells overexpress EVI1; (4) the overexpressing cells are mixed in with normal cells, as is the case in incipient human myeloid malignancies. While we have not documented EVI1 overexpression in specific lineages within the hematopoietic system in our mouse model, it is known that DOX induction of target genes harboring the tetracycline operons via the rtTA transactivator occurs in multiple compartments within the marrow, and, in general, gene activation in the marrow with this system is quite strong^16–18^. Thus, it is very likely that in our system, EVI1 overexpression occurs in both the HSC compartment, as well as more mature and differentiated derivatives. In humans with 3q26 rearrangements, a super-enhancer from the 3q21/*GATA2* locus relocates to be in proximity to *MECOM*^46^, resulting in high expression of EVI1^5^; whereas the EVI1 is exclusively active in long-term and short-term HSCs^4^, the *GATA2* gene is active both in HSCs and in more mature cells, including erythroid lineage cells^47^. It is known that 3q26 translocations in myeloid malignancies occur in early MPPs^48^, and leukemia-associated translocations have been found in erythroid cells and lymphoid cells^49^. Thus, it is very likely that in humans, the 3q26-rearranged chromosome is present and expressed as stem/progenitor cells mature to the MPP stage.

While binding of EVI1 to PU.1 has been noted previously^50^, the relationship between EVI1 and *Spi1*/PU.1 transcriptional regulation has not been investigated previously. Here, we document binding of EVI1 to a previously characterized regulatory element, the −14kbURE, together with EVI1-induced upregulation of PU.1. Strikingly, knocking down PU.1 abrogated myeloid skewing associated with EVI1 overexpression, revealing that PU.1 is necessary for EVI1-induced myeloid expansion. The mechanism by which EVI1 and PU.1 interfere with erythropoiesis likely includes antagonism of GATA1 function; PU.1 has been shown to have this activity^51,52^, and EVI1 is known to bind to GATA1’s DNA motif^22^, so it is possible that EVI1 itself, in addition to PU.1, impedes GATA1 function in developing erythroid cells.

The most striking transcriptional response induced by EVI1 is upregulation of Sca-1, which is known to have an important role in the granulocytic response to bacteria via TLR4, the receptor for lipopolysaccharides (LPS)^53,54^; PU.1 is known to play an important role in this^53^. Our data presented here indicate that upregulation of Sca-1 occurs via PU.1 action (Figs. 6, 7). The key findings of our work—that EVI1 overexpression causes lymphoid and erythroid suppression while expanding the myeloid compartment—bears a striking similarity to what is seen during emergency granulopoiesis^55^. Infection with a number of pathogenic organisms results in a shift away from erythropoiesis and lymphopoiesis and towards myelopoiesis^56–60^. The similarities between the effects of EVI1 on the hematopoietic system and the marrow’s response to infection suggest either that EVI1 is acting through molecular mimicry, or that it plays a role in the bacterial response. While EVI1 is normally only expressed in the long- and short-term HSCs^4^, recent findings indicate that it is also induced by LPS (E.A., A.S.P., unpublished findings; ref. ^61^); and a recently described mutation in EVI1 is susceptible to bacterial infections^62^.

In summary, our findings offer an insight into mechanisms of leukemogenesis, whereby a single nuclear oncoprotein can induce clinically significant suppression of erythropoiesis and lymphopoiesis, while promoting a shift in differentiation that results in a preleukemic expansion of myeloid cells. These findings help to explain the singular association between EVI1 and myeloid malignancies.

## Methods

### Mice

The *Evi1*^*TO*^ allele was generated by insertion of the NSTO cassette^63^ via homologous recombination in ES cells using standard techniques^64^. PU.1^gfp^ reporter mice^37^ and the *Spi-1*^*UREd*^ mice^40^ have been described. UBC-GFP mice were obtained from Jackson Labs. All procedures involving mice were approved by the institutional animal care and use committees of all participating institutions.

### Harvesting bone marrow

Bone marrow was harvested by one of two methods: (1) flushing the leg long bones the marrow space with flow cytometry (FACS) buffer (PBS, 2% fetal bovine serum (FBS)) using a 1 ml syringe and 25-gauge needle or (2) grinding femurs, tibia, and pelvis bones (2F2T2P) in FACS buffer using a mortar and pestle^65^.

### Flow cytometry and imaging flow cytometry

Marrow harvest, analysis, and transplantations and other procedures were performed as described^4,15,65,66^. Imaging flow was done on ImageStreamX with IDEAS 6.0 (Amnis). Accuri C6, FACSCalibur, LSR II, and FACScan instruments (BD Biosciences) were used for flow cytometric analysis and cell sorting. Supplementary Table 2 has cell-surface marker phenotypes. Cells were stained with antibodies targeted against the appropriate hematopoietic marker for 30 min on ice in the dark using manufacturer’s recommended antibody dilutions. Cells were then rinsed once with PBS and resuspended in 1× PBS, 2% FBS before analysis. Antibodies used for flow cytometic analysis and cell sorting are listed in Supplementary Table 3. Collection of all flow cytometry data were done using BD FACSDIVA software. Analyses of all flow cytometry data were done using FlowJo version 10 software (TreeStar).

### Proliferation and apoptosis

Mice were injected with 2 mg of BrdU 60 min before harvest; incorporation levels were assessed using kit (Cat#552598; BD Biosciences). Apoptosis rates were determined by flow with anti-annexinV (BD Biosciences).

### Cell lines and cell culture

EML cells were provided by Shickwann Tsai and were cultured as described^26^. Methylcellulose colony-forming assays for myeloid progenitors (M, G, and GM-CSF combined) were carried out by plating 10,000 bone marrow cells as previously described^67^. Other cell culture techniques were adapted from previously published protocols^4,66^.

### Genetic analysis

RT-qPCR, ChIP, and genomic PCR were performed as described^4,38,66^. The corresponding primers are listed in Supplementary Table 4.

### Statistical analysis

Data were analyzed using Prism (GraphPad); significance was determined using Student’s *t*-test (*α* = 0.05).

## Electronic supplementary material

Supplementary Information

## Data Availability

The authors declare that the data supporting the findings of this study are available within the paper and its supplementary information files. All other relevant data supporting the findings of this study are available from the corresponding author on reasonable request. ChIP-seq data are available in Figshare Repository with the identifier 10.6084/m9.figshare.7043468.
