# Supplementary material for: EVI1 overexpression reprograms hematopoiesis via upregulation of Spi1 transcription
Source: Nat Commun. 2018 Oct 12;9:4239. doi: 10.1038/s41467-018-06208-y (PMC6185954; doi:10.1038/s41467-018-06208-y)
Supplement: Supplementary file 1 — Supplementary Information [file 41467_2018_6208_MOESM1_ESM.pdf]

Supplementary Information

EVI1 overexpression reprograms hematopoiesis via upregulation of *Spi1* transcription

Ayoub et al.

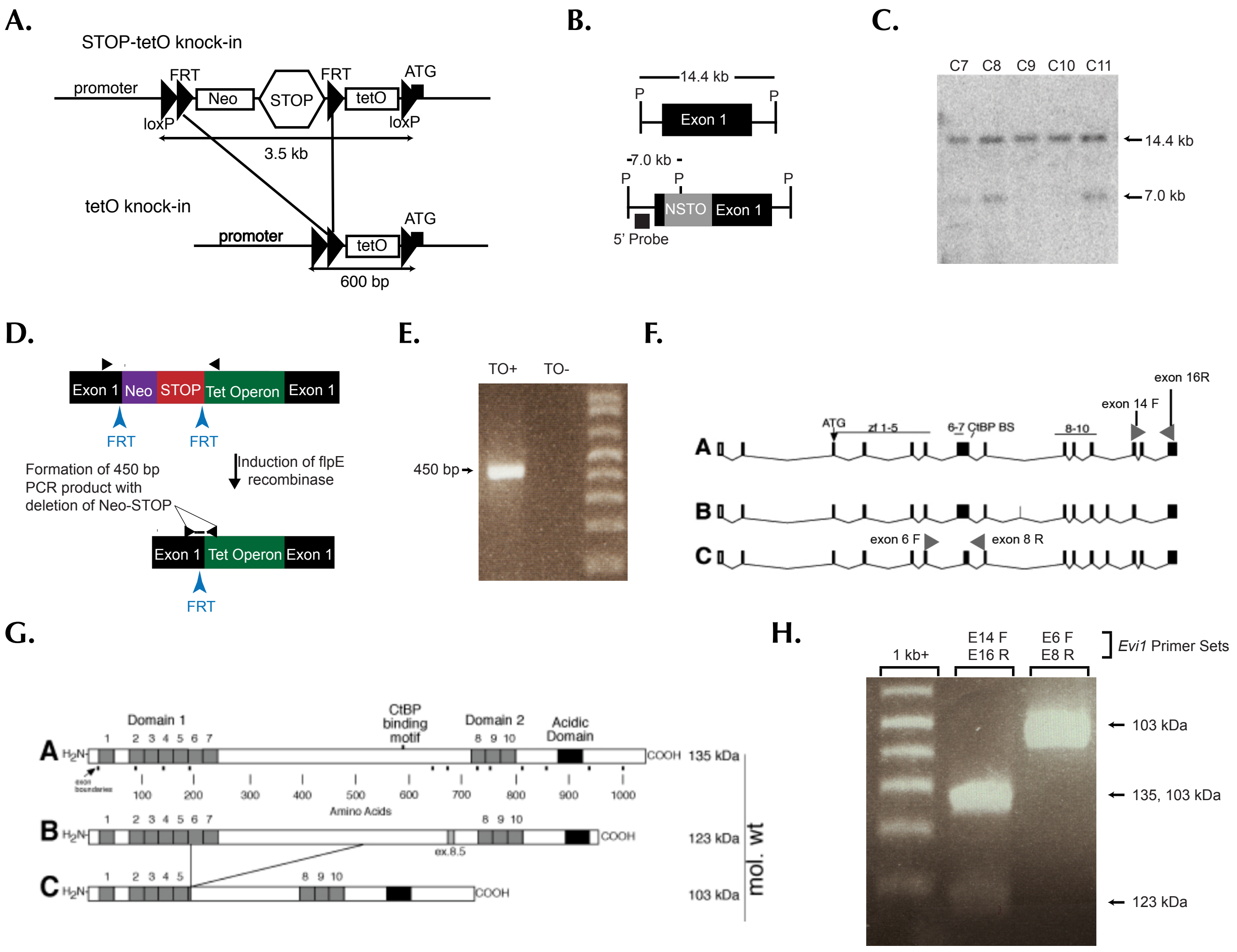

Supplementary Figure 1 | A. Diagram of the knock-in cassette for the creation of the Evi1TO allele. **B.** Schematic showing the expected sizes with a PvuII digest, using a 5′ hybridization probe. P denotes PvuII restriction enzyme sites. **C.** Southern blot of DNA from embryonic stem cell clones, digested with PvuII and hybridized with the 5′ probe. Clones C7, C8, and C11 show the presence of the rearranged allele. **D.** Schematic depicting the removal of the Neo-Stop cassette using FlpE deleter strain, B6.129S4-*Gt(ROSA)26*$^{Sortm1(FLP1)Dym/RainJ}$. **E.** PCR analysis of tail DNAs from the offspring from the cross with the FlpE deleter strain, using primers depicted in panel D, with a product size (450 bp) consistent with the loss of the Neo cassette. **F.** Diagram of the three major alternatively spliced *Evi1* transcripts, as well as location of the initiator methionine ATG, the locations of the encoded zinc fingers (zf 1-5, 6, 7, 8-10) and the CtBP interaction domain. Also shown is the loca- tion of the exon 6F and exon 8R primers for distinguishing splice form C from the other two; and primers 14F and 16R for distinguishing the B splice form from the other two. **G.** Diagram of the proteins encoded by the three alternatively spliced isoforms, with the zinc finger, CtBP binding, and acidic domains indicated. The apparent molecular weight of each isoform, in kilodaltons, is displayed to the right. **H.** PCR results from the analysis of cDNA prepared from RNA isolated from bone marrow of DOX-induced *Evi1*^{TO}, *R26*^{rtTA} mice, with primer pairs for detection of the B splice pattern (forward primer 14F, reverse primer 16R, yielding a band indicative of the presence of the B splicing pattern (denoted "123" to the right)) and the C splicing pattern (forward primer 6F, reverse primer 8R, yielding a band indicative of the C splicing pattern, denoted by "103" to the right).

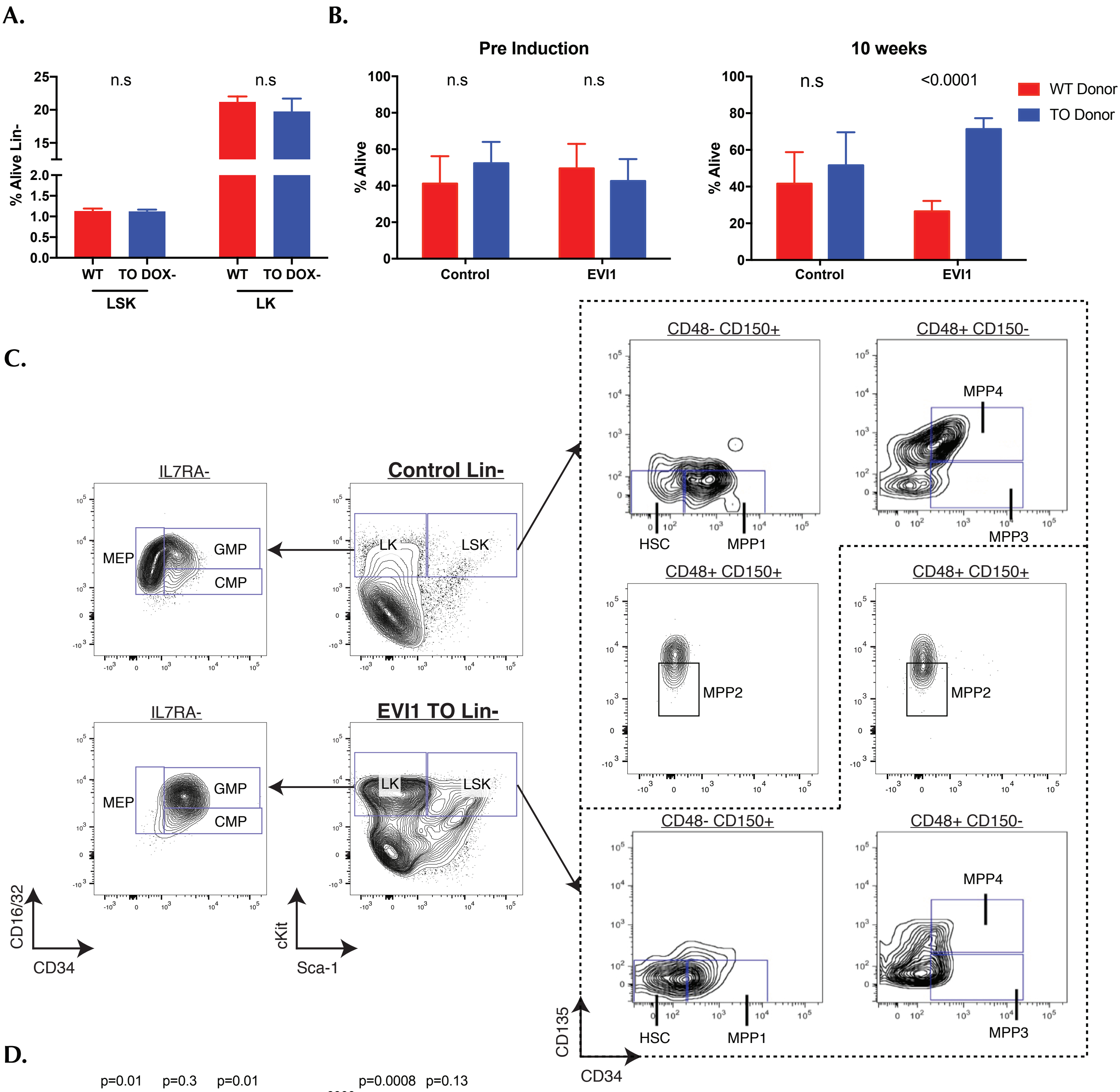

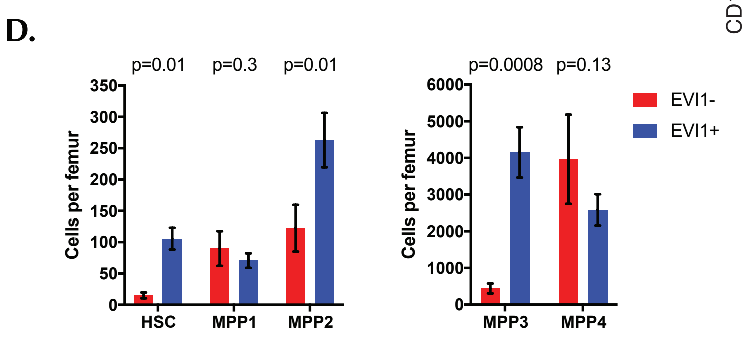

Supplemenary Figure 2 | **A.** Quantification of stem cell and progenitor populations LSK (Sca-1+/c-kit+ lineage-) and LK (Sca-1-/c-kit+ lineage-) using flow cytometry analysis in bone marrow from WT or uninduced (DOX-) *Evi1*$^{TO/TO}$ *Rosa26*$^{rtTA/rtTA}$ mice (n=3). **B-Left.** The ratio of WT to *Evi1*^{TO} cells in transplanted recipients after stable engraftment (4 weeks post transplantation) and prior to addition of DOX (n=4). **B-Right.** The ratio of WT to *Evi1*^{TO} cells in transplanted recipients 10 weeks after the addition of DOX (n=4). **C.** Representative FACS plots detailing the flow cytometry strategy that we adapted from Cabezas-Wallscheid et al.[1]. MPP populations within the LSK (Sca-1+/c-kit+ lineage-) compartment are quantified using cell surface markers (CD48, CD150, CD135, CD34). Progenitor populations (CMP, GMP, MEP) within the LK (Sca-1-/c-kit+ lineage-) compartment are quantified using cell surface markers (CD16/32, CD34) (n=3). **D.** Quantification of progenitor populations within LSK (Sca-1+/c-kit+ lineage-) in *Evi1*$^{TO/TO}$ *Rosa26*$^{rtTA/rtTA}$ mice at 3 days post-induction (n=3).

A.

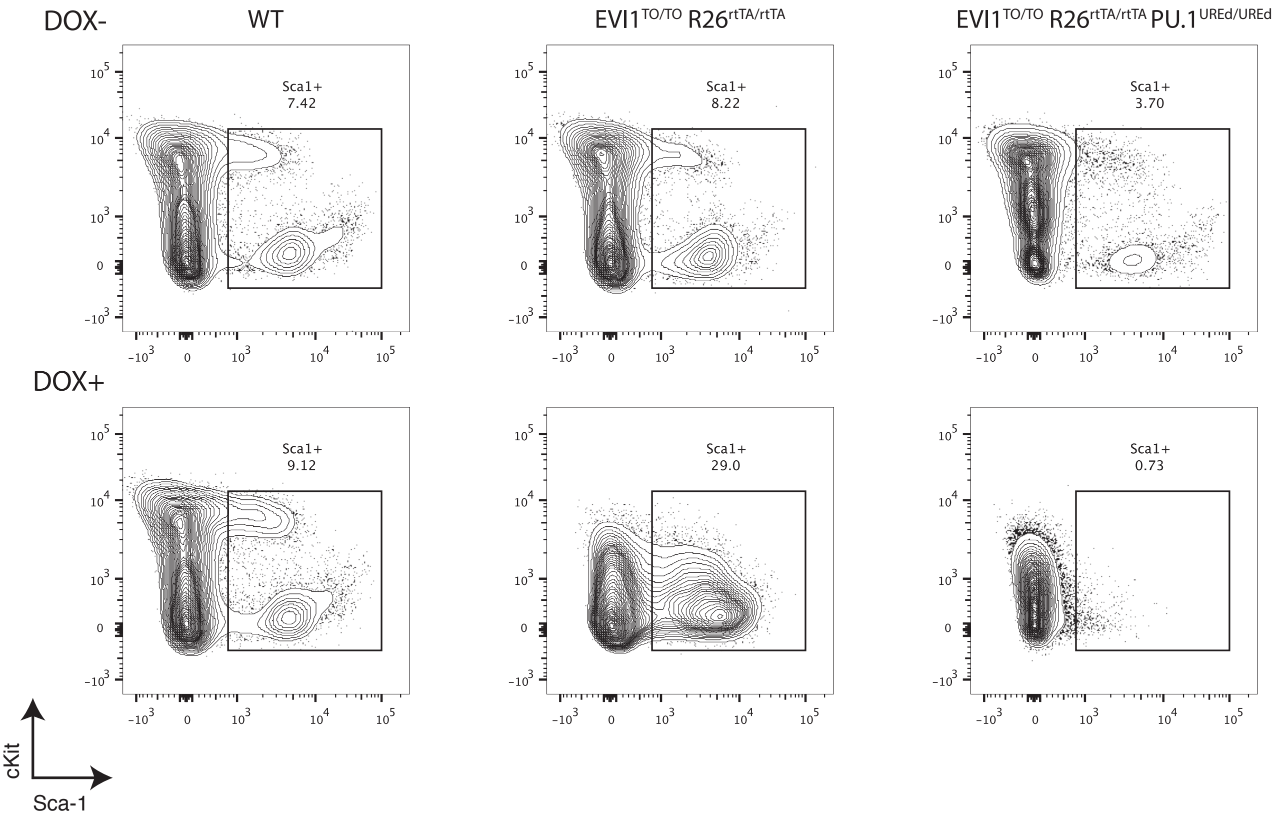

Supplementary Figure 3 | A. Representative cytometric analysis for Sca-1 and c-Kit, as indicated, of WBM from mice either without (top row) or with (bottom row) DOX treatment for 72 hrs. Three genotypes of mice were analyzed, as indicated on the top of each column. The percentage of Sca-1+ cells within Lin-/Alive cell population is indicated above the Sca-1 gate within each scatterplot (n=3).

Supplementary Table 1

Progenitor Compartment	Cell Surface Phenotype
HSC	LSK CD34- CD135- CD48- CD150+
MPP1	LSK CD34+ CD135- CD48- CD150+
MPP2	LSK CD34+ CD135- CD48+ CD150+
MPP3	LSK CD34+ CD135- CD48+ CD150-
MPP4	LSK CD34+ CD135+ CD48+ CD150-

Progenitor Compartment	Cell Surface Phenotype
CMP	LK CD34+ CD16/32^{low}
GMP	LK CD34+ CD16/32^{high}

Hematopoietic progenitors were analyzed by flow cytometry using the surface marker phenotype described and detailed by Cabezas- Wallscheid et al.[1]. Red text is used to highlight the differences in expression of surface markers that are used to identify each progenitor population.

Supplementary Table 2 | Cell surface markers phenotypes

Figure	Panel
2A	GFP, TER119=PerCP-Cy5.5, DAPI
2B,C,D	GFP, CD71-PE, CD41-PEcy7, Ter119-APC, DAPI
2E	GFP, TER119=PerCP-Cy5.5, Annexin-V=APC, 7AAD
2F	TER119=PerCP-Cy5.5, CD71=FITC, c-KIT=PE-Cy5, DAPI
2G	TER119=PerCP-Cy5.5, CD71=FITC, c-KIT=PE-Cy5, Anti-BrdU=APC, DAPI
3A	GFP, B220=PE-Cy7, DAPI
3B	GFP, CD3e-CFC594, CD11b-PEcy7, CD19-PerCPcy5.5, Gr1-APC-eF780, DAPI
3C	GFP, CD3e=PE-CF594, B220=PE-Cy7, Annexin-V=APC, 7AAD
3D	CD3e=PE-Cy7, CD4=PE, CD8a=APC, DAPI
3E	CD3e=PE-Cy7, CD4=PE, CD8a=APC, Annexin-V=FITC, DAPI
3F	CD3e=PE-Cy7, CD4=PE, CD8a=APC, Anti-BrdU=FITC, DAPI
4A	GFP, CD11b=V450, DAPI
4B	GFP, LY6G/C=APC-Cy7, DAPI
4C	GFP, cKit- PE-Cy5, Sca-1-PerCP-Cy5.5, CD16/32-APC-Cy7, CD34-AF700, CD127-BUV737, DAPI
4E	GFP, CD11b-PECF594, Gr1-APC-eF780, F4/80-PE, DAPI
4F	GFP, LY6G/C=APC-Cy7, Annexin-V=APC, 7AAD
5C	CD45.2-APC, CD11b=V450, B220=PE-Cy7, TER119=PerCP-Cy5.5, DAPI
6B	Sca-1-PerCP-Cy5.5, DAPI
6D	CD45.1-PE, CD45.2-APC, cKit- PE-Cy5, Sca-1-PerCP-Cy5.5, CD34-AF700, CD16/32-FITC, CD127-BUV737, (lineage depletion with B220, CD3e, Ter119, CD11b, Gr1), DAPI
6E	cKit- PE-Cy5, Sca-1-PerCP-Cy5.5, CD150-APC, CD48-APC-eFluor780, (lineage depletion with B220, CD3e, Ter119, CD11b, Gr1), DAPI
6F,G,H	GFP, Sca-1=PerCP-Cy5.5, DAPI
7E	(CD3e=Biotin, B220=Biotin Ter119=Biotin, Gr1=Biotin) Lin=Biotin PE-CF594 Streptavidin, c-KIT=PE-Cy5, Sca-1=PerCP-Cy5.5, DAPI
S.2A	GFP, (CD3e=Biotin, B220=Biotin Ter119=Biotin, Gr1=Biotin) Lin=Biotin PE-CF594 Streptavidin, c-KIT=PE-Cy5, Sca-1=PerCP-Cy5.5, DAPI
S.2B	GFP, DAPI
S.2C	CD16/32-APC-Cy7, CD34-AF700, cKit- PE-Cy5, Sca-1-PerCP-Cy5.5, CD135-PE, CD150-APC, CD48-APC-eFluor780, (lineage depletion with B220, CD3e, Ter119, CD11b, Gr1), DAPI
S.2D	Refer to Supplementary Figure 2C, and Supplementary Table 1
S.3A	(CD3e=Biotin, B220=Biotin Ter119=Biotin, Gr1=Biotin) Lin=Biotin PE-CF594 Streptavidin, Sca-1=PerCP-Cy5.5, DAPI

Supplementary Table 3 | Flow cytometry reagents

Reagent	Fluorescence	Source	Catalog Number
7AAD		BD Bioseceinces	559925
Annexin V	FITC	BD Bioseceinces	556547
Annexin V	APC	eBioscience	88-8007-72
Anti-Mouse Ig Particles		BD Bioseceinces	552843
Anti-Rat/Hamster Ig Particles		BD Bioseceinces	552845
Biotin Mouse Lineage Panel		BD Bioseceinces	559971
BrdU Flow Kit	APC	BD Bioseceinces	552598
CD117 (c-Kit)	PE-Cy5	eBioscience	15-1171-82
CD11b	V450	BD Bioseceinces	560455
CD11b	PE-CF594	BD Bioseceinces	562317
CD11b	PE-Cy7	eBioscience	25-0112-82
CD127	BUV737	BD Bioseceinces	564399
CD135 (Flt3)	PE	eBioscience	12-1351-82
CD150	APC	eBioscience	17-1501-81
CD16/32	APC-Cy7	BD Bioseceinces	560541
CD19	PerCP-Cy5.5	eBioscience	45-0193-82
CD34	Alexa Fluor 700	BD Bioseceinces	560518
CD3e	PE-CF594	BD Bioseceinces	562286
CD41	PE-Cy7	eBioscience	25-0411-82
CD45.1	PE	BD Bioseceinces	553776
CD45.2	APC	BD Bioseceinces	558702
CD45R/B220	PE-Cy7	BD Bioseceinces	552772
CD48	APC-eFluor 780	eBioscience	47-0481-82
CD71	PE	eBioscience	11-0711-82
CD71	FITC	BD Bioseceinces	553266
DAPI		Invitrogen	D3571
F4/80	PE	eBioscience	12-4801-80
Gr1 (Ly-6G/C)	APC-eFluor 780	eBioscience	47-5931-80
Ly-6A/E (Sca-1)	PerCP-Cy5.5	eBioscience	45-5981-82
Ly-6C	APC-Cy7	BD Bioseceinces	560596
Ly-6G	APC-Cy7	BD Bioseceinces	560600
Mouse B Lymphocyte Subset		BD Bioseceinces	558332
Mouse T Lymphocyte Subset		BD Bioseceinces	558431
PE-CF594 Streptavidin		BD Bioseceinces	562318
TER-119	APC	eBioscience	17-5921-82
TER-119	PerCP-Cy5.5	BD Bioseceinces	560512

Supplementary Table 4 | PCR primers

Mds1 Exon 2 F	TCCCTGATGACATCCCTATTCC
Evi1 Exon2 Rev	CATCTATGCAGAACTTCACATTGC
Evi1 Exon 1 F (70)	CTGAGTTGAGGCCGTAGAAATC
Cebpe R	CGCTCGTTTTCAGCCATGTA
Cebpe F	AAGGCCAAGAGGCGCATT
Cebpg R	GTATCTTGAGCTTTCTGCTTGCT
Cebpg F	GCGCAGAGAGCGGAACAA
Gapdh RT F	TGCACCACCACTGCTTAG
Gapdh RT R	GGATGCAGGGATGATGTTC
Gfi1b F	CAGGGACAGTGTGGAGGTTC
Gfi1b R	CTAGAAAGGACCGTGGCATT
Lmo2 F	ATGTCCTCGGCCATCGAAAG
Lmo2 R	CGGTCCCCTATGTTCTGCTG
Spi1 R	TGACTACTACTCCTTCGTGG
Spi1 F	GATAAGGGAAGCACATCCGG
rtTA (A)	AAAGTCGCTCTGAGTTGTTAT
rtTA (B)	AAGACCGCGAAGAGTTTGTC
rtTA (C)	GGAGCGGGAGAAATGGATATG
EVI1-TO F	AAAGTCGCTCTGAGTTGTTAT
EVI1-TO R	AAGACCGCGAAGAGTTTGTC

Note: rtTA primers: A+B for rtTA allele, 320 bp; B+C for WT allele, 600 bp.

Supplementary References

1 Cabezas-Wallscheid, N. *et al.* Identification of Regulatory Networks in HSCs and Their Immediate Progeny via Integrated Proteome, Transcriptome, and DNA Methylome Analysis. *Cell Stem Cell* **4**, 507-522 (2014).
